# Myocarditis after Trimethoprim/Sulfamethoxazole Treatment for Ehrlichiosis

**DOI:** 10.3201/eid1912.121459

**Published:** 2013-12

**Authors:** Seema U. Nayak, Gary L. Simon

**Affiliations:** Johns Hopkins University School of Medicine, Baltimore, Maryland, USA (S.U. Nayak);; George Washington University Medical Center, Washington, DC, USA (G.L. Simon)

**Keywords:** Ehrlichia, myocarditis, trimethoprim/sulfamethoxazole, bacteria, ehrlichiosis, zoonoses, tickborne, ticks

## Abstract

The manifestations of human monocytic ehrlichiosis range from a mild febrile syndrome to a severe multisystem illness. Myocardial involvement is uncommon. We report a woman, 78 years of age, who was treated with trimethoprim/sulfamethoxazole after a tick bite, in whom myocarditis was subsequently diagnosed. She recovered completely after doxycycline therapy.

## Abstract 2

Myocarditis after Treatment for Ehrlichiosis

Human monocytic ehrlichiosis (HME) is caused by an obligate intracellular gram-negative bacteria of the family *Anaplasmataceae*. The first reported case of ehrlichiosis occurred in a man, 51 years of age, in Arkansas, United States. The man experienced a prolonged febrile illness after being bitten by ticks. Four years later, *Ehrlichia chaffeensis*, the causative agent of this syndrome, was recognized (*1*). Ticks acquire *E. chaffeensis* from a reservoir host, the white-tailed deer, and transmit the organism to humans during blood meals (*2*).

The clinical manifestations of HME range from a mild febrile syndrome to severe multisystemic illness. Although ehrlichiosis has sometimes been referred to as Rocky mountain spotted fever without the rash, a rash is not uncommon (*2*). Gastrointestinal, pulmonary, and central nervous system symptoms are well described, but cardiovascular disease is rare (*2*). Including the patient described herein, 4 persons in whom HME was diagnosed have had myocardial involvement.

**The Study**

A woman, 78 years of age, who had a 4-day history of fevers, chills, fatigue, and myalgias sought medical treatment after a syncopal episode. She denied dyspnea or chest pain. Ten days earlier, while in southern Virginia, she had noticed a tick on her ankle. After tick removal, the area became inflamed, and the patient received trimethoprim/sulfamethoxazole (TMP/SMX) from a local urgent care center.

Her vital signs were as follows: oral temperature 39.3°C, blood pressure 120/60 mm Hg, heart rate 90 bpm, respiratory rate 16 breaths per min, and oxygen saturation 98% on room air. Except for a 2–cm erythematous patch on the medial aspect of her right ankle, results of her physical examination were normal. Her leukocyte count was 1.4 ×10^3^ cells/μL (34% non-segmented neutrophils) and platelet count was 66 × 10^3^/μL. Blood cultures were negative. The serum aspartate aminotransferase level was 173 U/L (reference <50 U/L), and the serum alanine aminotransferase level was 109 U/L (reference <50 U/L). Urinary sediment was unremarkable and a chest radiograph was normal. 

The following day, the patient became dyspneic. A room air arterial blood gas revealed a pH of 7.43 (reference range 7.38–7.42), PO_2_ of 70 mm Hg (reference range 75–100 mm Hg), and PCO_2_ of 20 mm Hg (reference range 38-42 mm Hg). Pulmonary congestion without focal infiltrates was evident on a chest radiograph; an electrocardiogram indicated normal voltage, and saddle-shaped S-T elevations in 6 leads did not indicate ischemic distribution. On hospital day 3, her cardiac enzyme levels were markedly elevated: creatine kinase was 2,524 U/L (reference <325 U/L), creatine kinase-MB 27.3 ng/mL (reference <2.3 ng/mL), and troponin 27.3 ng/mL (reference 0–0.03 ng/mLl). A transthoracic echocardiogram revealed left ventricular systolic dysfunction with global hypokinesis and an ejection fraction of 30% (reference range 55–70%). Cardiac magnetic resonance imaging showed mild atherosclerosis.

Review of the patient’s peripheral blood smear from the day of admission showed several monocytes with characteristic morulae. She was empirically treated with doxycycline, and experienced prompt defervescence. Her cardiac decompensation resolved over several days and an ejection fraction of 70% was noted on echocardiogram 10 weeks after her admission.

Serologic results for *Borrelia bergdorferi*, *Rickettsia rickettsii*, *Anaplasma phagocytophilum*, and *Francisella tularensis* were negative. Initial serologic analysis for *E. chaffeensis* demonstrated a positive result (IgM titer 320) and a negative IgG result . Convalescent-phase serologic analysis 1 month after discharge showed an IgG titer for *E. chaffeensis* of 8,192, indicating recent infection; markers of cardiac injury had returned to reference levels.

**Conclusions**

The Dallas criteria for a diagnosis of myocarditis are applied based on histopathologic findings of an inflammatory cellular infiltrate with or without myocyte necrosis. Unfortunately, these criteria have low sensitivity, lack prognostic value, and necessitate an invasive procedure. Autopsy studies have shown that myocardial inflammation is not homogeneous and that sampling issues can contribute to a high rate of false negative endomyocardial biopsies. In clinical practice, the diagnosis is made based on the clinical syndrome, cardiac biomarkers, and electrocardiographic and echocardiographic findings (*3*).

Although a wide variety of pathogenic organisms have been associated with myocarditis, the etiology of this disorder in most patients remains idiopathic. In those cases in which an etiologic agent has been identified, viruses, in particular coxsackie B viruses, have been most frequently implicated (*4*). Endomyocardial biopsies during the late 1990s revealed other viral agents such as adenovirus, influenza A and B, cytomegalovirus, parvovirus B-19, and Epstein-Barr virus, among others (*3*,*4*). Myocardial involvement and cardiac dysfunction are frequently recognized in patients with HIV infection.

*Trichinella spiralis* and *Toxoplasma gondii* have also been recognized as causes of myocarditis, and, outside the United States, trypanosomes are commonly recognized etiologic agents (*4*). Bacteria can cause myocarditis through the effects of toxins, as is the case with diphtheria. Endotoxins have a direct suppressant effect on myocardial contractility, and myocardial dysfunction can occur in patients infected with gram-negative bacteremia. Alternatively, the presence of other organismsa, such as *Mycoplasma pneumoniae*, *Chlamydophilia* spp., or *Borrelia bergdorferi* within the myocardium has been demonstrated, indicating a more direct effect (*3*,*4*).

Myocardial involvement is a rare complication of HME. Studies in which dog models were used have shown that acute infection with *E. canis* is a risk factor for myocardial injury (*5*). In 2 earlier reported cases of HME with myocardial involvement, previously healthy men had clinical symptoms of HME confirmed by serologic analysis and left ventricular dysfunction and electrocardiographic abnormalities developed, similar to those of the patient in this study. Myocarditis resolved in both patients after doxycycline therapy (*6*,*7*). A third case of HME-related myocarditis occurred in a patient with Wegener granulomatosis (*8*). The patient was immunosuppressed by use of a tumor necrosis factor-α inhibitor, which may have contributed to the more severe manifestation of HME. This patient also appeared to recover after initiation of doxycyline. Jahangir et al. reported the sudden death of an otherwise healthy outdoor worker 19 days after the worker was bitten by a tick. An autopsy revealed transmural myocarditis and pulmonary congestion and serology consistent with human granulocytic anaplasmosis (*9*).

There is a demonstrated association between use of trimethoprim/sulfamethoxazole and fulminant manifestation of rickettsial diseases; the mechanism is unknown. Severe cases of HME have been reported in otherwise healthy adolescents taking short courses of trimethoprim/sulfamethoxazole (*10*,*11*), and in transplant patients receiving long-term regimens of sulfa drugs as prophylaxis (*12*,*13*). Our patient’s disease severity may have been exacerbated by her recently prescribed regimen of trimethoprim/sulfamethoxazole.

Myocarditis associated with human monocytic ehrlichiosis is distinctly uncommon. The possibility that this condition is caused by simultaneous infection with another microorganism cannot be excluded, although such dual infection is rare (*14*). Whether subclinical myocarditis may occur more frequently is unknown, but increased evaluation of cardiac function may reveal that this is a more common phenomenon.

The mechanism whereby this organism produces transient myocardial dysfunction is unknown. Rickettsial infection is characterized by direct endothelial cell infection and inflammation mediated by cytokine and chemokine activity, which leads to increased microvascular permeability (*15*). Occasionally, small vessel occlusion and local ischemia may occur. In patients with ehrlichiosis, perivascular lymphohistiocytic infiltrates may be seen on histopathologic examination (*16*). 

The observed improvement in myocardial function following doxycycline therapy in the patient in this study and in those previously described suggests that myocardial dysfunction may be caused by direct infection of the myocyte or a toxic effect of sulfa drugs, or may be secondary to an innate inflammatory response mediated by *E. chaffeensis*. It would be less likely to be a result of the host’s adaptive immune response, e.g., rheumatic fever. Considering the spread of this organism throughout the south central and southeastern United States, and the frequent use of trimethoprim/sulfamethoxazole to treat localized soft-tissue infections, it is necessary to recognize this pathogen as a treatable cause of myocarditis.
